# The mediating effects of gestational diabetes mellitus and hypertensive disorders of pregnancy between maternal advanced age, previous caesarean section and the risk of small- or large-for-gestational-age newborns: a multicentric prospective cohort study in southern China

**DOI:** 10.7189/jogh.15.04053

**Published:** 2025-01-03

**Authors:** Lihua Lin, Bin Sun, Xiaomei Wang, Ronghua Zhang, Juan Lin, Jianying Yan

**Affiliations:** 1 Department of Healthcare, Fujian Maternity and Child Health Hospital, Affiliated Hospital of Fujian Medical University, Fuzhou, Fujian, China; 2 Division of Birth Cohort Study, Fujian Maternity and Child Health Hospital, Affiliated Hospital of Fujian Medical University, Fuzhou, Fujian, China; 3 Department of Obstetrics, Fujian Maternity and Child Health Hospital, Affiliated Hospital of Fujian Medical University, Fuzhou, Fujian, China; † Joint senior authorship.

## Abstract

**Background:**

Maternal obstetric characteristics have a key role in determining the occurrence of pregnancy-related disorders and subsequent neonatal outcomes. We aimed to investigate the mediating impact of gestational diabetes mellitus (GDM) and hypertensive disorder of pregnancy (HDP) on the relationship between maternal advanced age, previous caesarean section, and the risk of either large for gestational age (LGA) or small for gestational age (SGA) infants.

**Methods:**

We used data from a prospective multicentre cohort study conducted through China's National Maternal Near-miss Surveillance System from January 2012 to December 2021. We performed univariate and multivariate logistic regression analyses to examine the connections between maternal advanced age, previous caesarean section, GDM and HDP, and the risks of LGA and SGA, as well as mediation analyses to assess the mediating effect of GDM and/or HDP on the relationship between maternal advanced age, previous caesarean section, and the risks of LGA and SGA.

**Results:**

We included 482 458 women in our study, of whom13.5% were classified as advanced age, 51.4% as multipara, and 16.3% had a history of uterine scarring. Following adjustments for covariates, we found statistically significant associations between maternal advanced age and GDM (adjusted odds ratio (aOR) = 1.79; 95% confidence interval (CI) = 1.75, 1.83), maternal advanced age and HDP (aOR = 1.93; 95% CI = 1.86, 2.01), previous caesarean section and GDM (aOR = 1.13, 95% CI = 1.11, 1.16), previous caesarean section and HDP (aOR = 1.24; 95% CI = 1.20, 1.28), GDM and LGA (aOR = 1.32; 95% CI = 1.30, 1.35), and HDP and SGA (aOR = 3.93; 95% CI = 3.75, 4.12). The influence of maternal advanced age on SGA was significantly mediated by HDP, accounting for 68.96% of the mediation effect. Furthermore, GDM and HDP served as significant mediators in the relationship between previous caesarean section and the risks of LGA and SGA, with mediation proportions of 5.62% and 4.49%, respectively.

**Conclusions:**

We found HDP has a mediating role in the impact of maternal advanced age and previous caesarean section individually on SGA risk, while GDM acts as a mediator in the connection between previous caesarean section and LGA risk.

Birth weight is a crucial indicator of fetal health, with abnormal birth weight falling into two categories: being too large or too small. The proper fetal growth and development are related to several factors, notably those pertaining to the health of the mother. According to birth weight and gestational age, newborns are often classified as large for gestational age (LGA), appropriate for gestational age (AGA), and small for gestational age (SGA). Specifically, LAG and SGA are defined as a birth weight at or above the 90th percentile or below the 10th percentile, respectively, on the birth weight curve for a given gestational age and sex [1]. Previous reports indicate that the prevalence of LGA and SGA to be at 22.1% and 6.5% in China, respectively [2,3]. Both LGA and SGA are considered as adverse neonatal outcomes due to the associated short- and long-term health complications. Moreover, LGA births face an increased risk of complications such as shoulder dystocia, nerve injuries, neonatal hypoglycaemia, and birth asphyxia for short-term outcomes [4-7], while also being predisposed to long-term issues like obesity, adverse metabolic outcomes and cardiovascular problems in later life [8–11]. Meanwhile, SGA newborns are usually predisposed to hypoglycaemia, polycythaemia, and higher mortality and morbidities rates (nearly 1.7- to 4-fold higher compared with AGA), especially if born prematurely [12–14]. Both LGA and SGA births contribute to increased medical cost and diminished economic productivity over the long term. Given the potential risks associated with LGA and SGA births, researchers and obstetricians have begun more actively focussing on prevention strategies [15,16]. Early identification of fetuses at risk of LGA and SGA, coupled with appropriate prenatal care and delivery management, can effectively reduce the incidence of perinatal complications during labour. Various known risk factors contribute to high birth weight, including conditions such as gestational diabetes mellitus (GDM), high pre-pregnancy body mass index, multiparity, advanced maternal age, and excessive weight gain during pregnancy [17–19]. Similarly, factors influencing the occurrence of SGA infants include advanced maternal age, nulliparity, and hypertensive disorders of pregnancy (HDPs) [20–25].

Conditions such as GDM and HDPs significantly impact maternal health and fetal development. For example, GDM affects more than 1 in 13 pregnancies and is associated with higher risk of LGA and other neonatal morbidity [26,27], while HDPs occur among approximately 10% of pregnancies and affects functional damage of organs as well as affects intrauterine growth and fetal development [28,29]. In contrast to GDM, however, HDPs are associated with increased risk of SGA [30]. Maternal obstetric characteristics play a key role in determining the occurrence of pregnancy-related disorders and subsequent neonatal outcomes. A study on the obstetrical characteristics on complications and adverse outcomes in southwestern China showed that women with advanced age are more likely to development preeclampsia and GDM [31]. In their cohort study, Khalil et al. [32] found that advanced maternal age, when analysed both as a continuous and as a categorical variable in regression analyses, is associated with an increased risk of pre-eclampsia, SGA, and GDM.

The high primary caesarean section rate in China means that more women have a previous caesarean section and is an important risk factor for placental problems and other pregnancy disorders [33–35]. Research has suggested that previous caesarean section also raise the likelihood of preterm birth, SGA, and low birth weight [36]. In light of changing fertility policies in China and the associated shifts in maternal obstetric characteristics, including advanced maternal age and a higher proportion of women with previous caesarean sections, there is a need to understand the relationship between these factors and the risks of LGA and SGA among Chinese women [37,38], as well as the potential mediating role of GDM and HDP in the relationship between maternal obstetric characteristics and infant growth.

Considering this, we aimed to quantify the effect of maternal advanced age and previous caesarean section and the mediating effects of GDM and HDP on the risks of LGA and SGA.

## METHODS

### Data source and study population

We obtained data from China’s National Maternal Near-miss Surveillance System (NMNMSS). Established in October 2010, the NMNMSS encompasses 14 hospitals in Fujian province and builds a patient-based database from individual survey forms. These forms gather data on various aspects including sociodemographic characteristics, obstetric history, method of delivery, pregnancy outcomes, and complications [39]. Women who are hospitalised during pregnancy or postpartum period with a termination of pregnancy or with accompanying complications are included for data collection. The NMNMSS incorporates an automatic data quality control process to ensure data consistency and integrity upon submission. Moreover, multiple levels of quality control are implemented manually, including monthly self-inspections by hospital, semi-annual evaluations at the county level, and yearly assessments at the municipal, provincial, and national levels, all aimed at guaranteeing data accuracy. Any errors identified during quality control are rectified previous to resubmission.

For the purposes of this analysis, we extract data spanning the period from January 2012 to December 2021. As preeclampsia and GDM can occur after 24 weeks of gestation, we excluded women with a gestational age of less than 28 weeks and cases of stillbirth or embryonic death.

### Definition of variables

#### Exposure, outcomes, and mediator

We defined SGA and LGA as a birth weight less than 10th or more than 90th percentile of a standard birth weight based on gender and gestational age, respectively [40]. Maternal advanced age was defined as being 35 years or older at the time of delivery. Previous caesarean section referred to the occurrence of a caesarean delivery in past pregnancies.

The primary outcomes of our were SGA and LGA, while GDM and HDP were considered as potential influencing factors. Here, GDM was diagnosed based on the 75 g oral glucose tolerance test at 24–28 gestational age by the International Association of Diabetes and Pregnancy Study Groups (IADPSG) criteria as one or more of the following: fasting plasma glucose >5.1 mmol/L, 1 hour plasma glucose >10.0 mmol/L, or 2 hours glucose >8.5 [41], while HDP was defined as blood pressure ≥140/90 mm Hg, occurring after 20 weeks gestation but without proteinuria [42].

#### Covariates

We included the following potential covariates: education level (college or higher, completed high school, completed middle school, completed primary school, illiteracy or missing), marital status (married, unmarried, others), times of prenatal care (continuous variable), parity (nultipara or multipara), and gravidity (1, 2, and ≥3).

#### Missing data

We had some missing data on the women’s education level (1.1%) and times of antenatal examination (0.7%). In general, directly removing or deleting records containing missing data may introduce bias into a given dataset. As the proportion of missing data in our study was small (1.8%), we considered missing data as just another value for the categorical variable.

#### Statistical analysis

We summarised the maternal characteristics using means (x̄) and standard deviations (SDs) or numbers and proportions, and we compared them through analysis of variance or χ^2^ tests according to birth weight categories. We conducted univariate and multivariate logistic regression analyses to investigate the association between maternal advanced age, previous caesarean section, and risks of LGA and SGA, as well as maternal advanced age, previous caesarean section, and the likelihood of GDM and/or HDP. We also examined the connection between GDM and/or HDP and the risks of LGA and SGA using two models: the crude model (model 1) and another one adjusted for marital status, educational level, times of antenatal examination, parity, and gravidity (model 2). We calculated odds ratios (ORs) and 95% confidence intervals (CI) to assess these risks.

**Figure 1:**
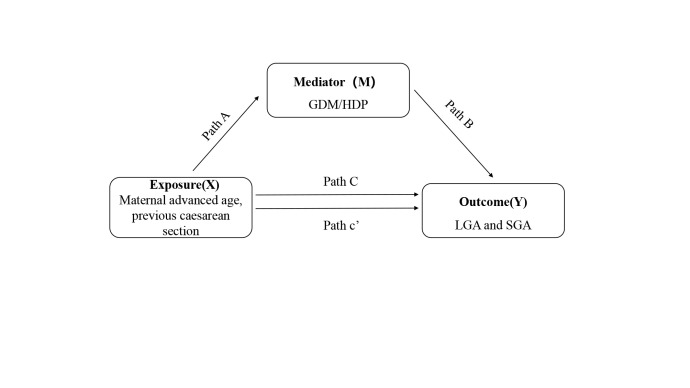
Total (Path C), direct (Path c’), and indirect (Path A and B) effects of the association between the exposure (X) and outcome (Y) via the mediator (M). GDM – gestational diabetes mellitus, HDP – hypertensive disorder of pregnancy, LGA – large for gestational age, SGA – small for gestational age.

**Figure 2:**
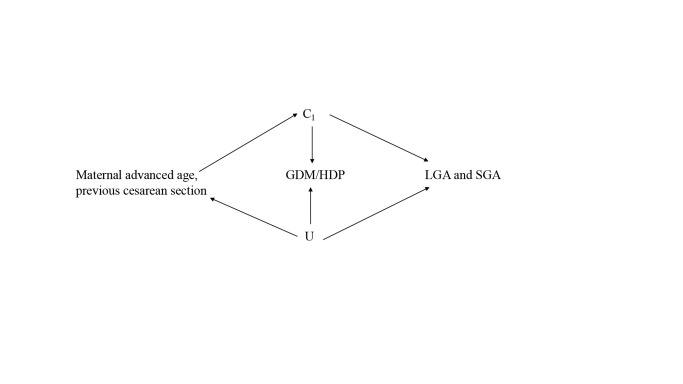
Directed acyclic graph for the association between maternal advanced age, previous caesarean section (exposure (X)) and LGA, SGA (outcome (Y)) mediated through GDM and HDP (mediator (M)). Observed confounders C1 are marital status, educational level, times of antenatal examination, parity and gravidity, which are potential common causes for X, M, and Y (unobserved confounders (U)). GDM – gestational diabetes mellitus, HDP – hypertensive disorder of pregnancy, LGA – large for gestational age, SGA – small for gestational age.

We performed mediation analyses using a SAS macro (%mediation) to investigate the mediating effect of GDM and/or HDP on the relationship between maternal advanced age, previous caesarean section, and the risks of LGA and SGA. Four assumptions were needed in the mediation analyses: no unmeasured confounder existed between exposure-outcome relationship; no unmeasured confounder existed between mediator-outcome relationship; no unmeasured confounder existed between exposure-mediator relationship; no mediator-outcome confounder was affected by the exposure. If these assumptions held, the direct effect referred to the effects of the maternal advanced age and previous caesarean section on LGA or SGA without any effect that occurs via the effect of maternal GDM and HDP. The indirect effect, meanwhile, was the effect of maternal GDM and HDP on LGA or SGA, independent of any effect of maternal advanced age and previous caesarean section. In this analysis, the total effect (95% CI), natural direct effect (95% CI), natural indirect effect (95% CI), and mediated proportion were present (**Figure 1**, **Figure 2**). Path A evaluated the impact of maternal advanced age and previous caesarean section on GDM and/or HDP. Path B assessed the impact of GDM and HDP on LGA or SGA. Path C represented the total effect of maternal advanced age and previous caesarean section on LGA and SGA. Path c’ referred to the direct effect, while Path A and Path B referred to the indirect effect. We adjusted all the estimates of mediating effect for marital status, educational level, times of prenatal care, parity and gravidity, and we also calculated the proportion mediated by GMD or HDP that contributed to the total effects.

For all statistical analyses, we considered *P* < 0.05 (two-tailed) as statistically significant. We conducted the analyses in SAS, version 9.3 (SAS Institute, Cary, NC, USA) and SPSS, version 21.0 (SPSS Inc., Chicago, IL, USA).

#### Ethical approval

We received approval from the ethics committee of the Fujian Maternity and Child Hospital (approval number: 2023KY154), which also waived the need for informed consent as we the data sourced from China's NMNMSS did not contain any personally identifiable information. All methodology in this study adhered to relevant guidelines and regulations outlined in the Declaration of Helsinki.

## RESULTS

### Characteristics of study population

In total, 489 584 women with singleton live births were reported in the NMNMSS during the study period. We excluded women with a gestational age of less than 28 weeks (n = 6639), cases of stillbirth or embryonic death (n = 487), leaving 482 458 eligible women for our final analysis. Among the participants, 13.5% were classified as being at an advanced age, 51.4% as multipara, and 16.3% had a history of uterine scarring. Overall, 23 958 women (5.0%) delivered SGA infants, while 78 028 women (16.1%) delivered LGA infants. Within the SGA group, 2637 women (11.0%) were diagnosed with GDM, compared to 13 151 women (16.9%) in the LGA group. In terms of HDP, 2575 women (10.7%) in the SGA group experienced this condition, while only 2680 women (3.4%) in the LGA group did (**Table 1**).

**Table 1:** Characteristics of selected participants, presented as n (%)

		Size for gestational age			
**Characteristics**	**Total, n = 482 458**	**AGA, n = 380 472 (78.9%)**	**SGA, n = 23 958 (5.0%)**	**LGA, n = 78 028 (16.1%)**	***P*-value**
Maternal age in years					<0.001
*<35*	417 499 (86.5)	331 410 (87.1)	21 057 (87.9)	65 032 (83.3)	
*≥35*	64 959 (13.5)	49 062 (12.9)	2901 (12.1)	12 996 (16.7)	
Education					<0.001
*College or higher*	187 303 (38.8)	149 822 (39.4)	7985 (33.3)	29 496 (37.8)	
*Completed high school*	86 852 (18.0)	67 945 (17.9)	4015 (16.8)	14 892 (19.1)	
*Completed middle school*	185 749 (38.5)	145 349 (38.2)	10 451 (43.6)	29 949 (38.4)	
*Completed primary school*	14 810 (3.1)	11 402 (3.0)	986 (4.1)	2422 (3.1)	
*Illiteracy*	2304 (0.5)	1736 (0.5)	236 (1.0)	332 (0.4)	
*Missing*	5440 (1.1)	4218 (1.1)	285 (1.2)	937 (1.2)	
Gravidity					<0.001
*1*	170 807 (35.4)	138 571 (36.4)	10 689 (44.6)	21 547 (27.6)	
*2*	152 795 (31.7)	120 692 (31.7)	6732 (28.1)	25 371 (32.5)	
*≥3*	158 856 (32.9)	121 209 (31.9)	6537 (27.3)	31 110 (39.9)	
Parity					<0.001
*Primiparous*	234 415 (48.6)	189 181 (49.7)	13 934 (58.2)	31 300 (40.1)	
*Multiparous*	248 043 (51.4)	191 291 (50.3)	10 024 (41.8)	46 728 (59.9)	
Times of antenatal examination					<0.001
*≤5*	132 332 (27.4)	103 966 (27.3)	8328 (34.8)	20 038 (25.7)	
*6–9*	171 342 (35.5)	134 956 (35.5)	8603 (35.9)	27 783 (356)	
*≥10*	175 204 (36.3)	138 761 (36.5)	6803 (28.4)	29 640 (38.0)	
*Missing*	3580 (0.7)	2789 (0.7)	224 (0.9)	567 (0.7)	
Previous caesarean section					<0.001
*Yes*	78 868 (16.3)	60 111 (15.8)	2799 (11.7)	15 958 (20.5)	
*No*	403 540 (83.6)	320 323 (84.2)	21 159 (88.3)	62 058 (79.5)	
GDM					<0.001
*Yes*	64 643(13.2)	47 855 (12.6)	2637 (11.0)	13 151 (16.9)	
*No*	418 815(86.8)	332 617 (87.4)	21 321 (89.0)	64 877 (83.1)	
HDP					<0.001
*Yes*	16 569 (3.4)	11 314 (3.0)	2575 (10.7)	2680 (3.4)	
*No*	465 889 (96.6)	369 158 (97.0)	21 383 (89.3)	75 348 (96.6)	

LGA – large for gestational age, GDM – gestational diabetes mellitus, HDP – hypertensive disorder of pregnancy, SGA – small for gestational age

Over one-third of the women in the LGA group had a high gravidity (three or more pregnancies), a proportion significantly higher than in the other two groups (*P* < 0.001). There were significant differences in the distribution of advanced age (*P* < 0.001), education level (*P* < 0.001), parity (*P* < 0.001), and history of caesarean section among the three groups (*P* < 0.001).

### Association between maternal advanced age, previous caesarean section, and risks of LGA and SGA

**Figure 3:**
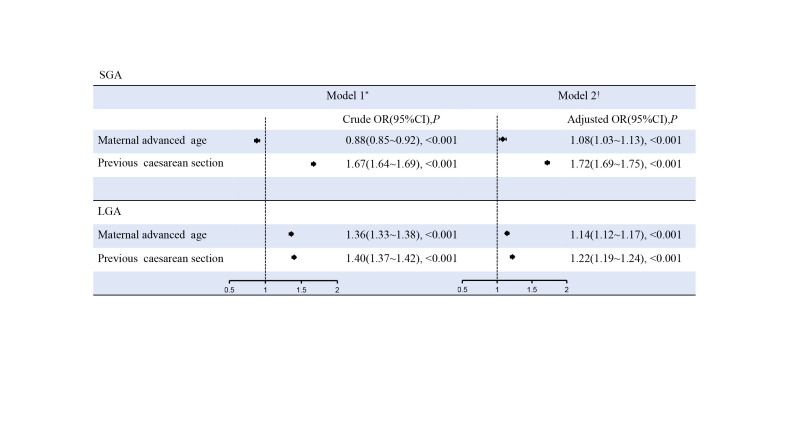
Association between maternal advance age, previous caesarean section and risks of LGA and SGA. *Model 1 was the crude model. †Model 2 was adjusted for marital status, educational level, times of prenatal care, parity and gravidity. CI – confidence interval, LGA – large for gestational age, OR – odd ratio, SGA – small for gestational age.

The results of our binary logistic regression analysis indicated that both maternal advanced age (OR = 1.08; 95% CI = 1.03, 1.13) and previous caesarean section (OR = 1.72; 95% CI = 1.69, 1.75) were high-risk factors for SGA in the adjusted models (**Figure 3**). We also observed significant associations between maternal advanced age and LGA (OR = 1.14; 95% CI = 1.12, 1.17), as well as previous caesarean section and LGA (OR = 1.22; 95% CI = 1.19, 1.24).

### Association between maternal advanced age and previous caesarean section and GDM or HDP, GDM or HDP and risks of LGA and SGA

**Figure 4:**
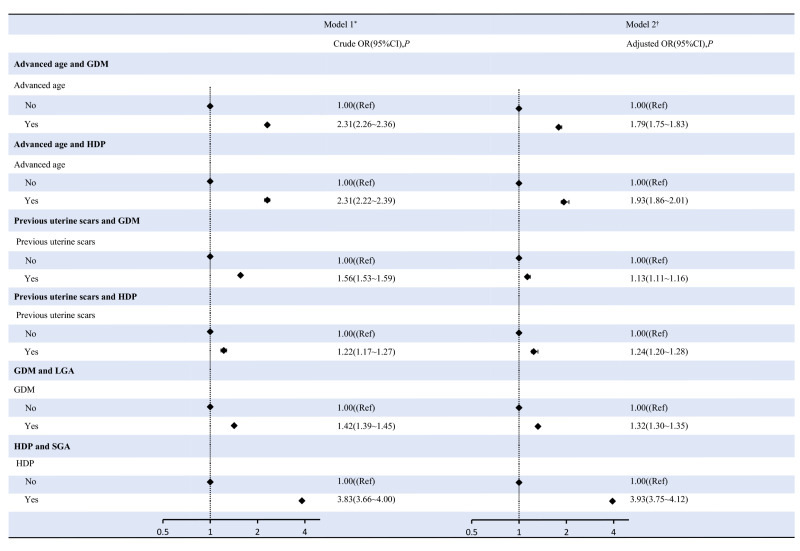
Association between maternal advanced age, previous caesarean section and GDM or HDP, GDM or HDP and risks of LGA and SGA. *Model 1 was the crude model. †Model 2 was adjusted for marital status, educational level, times of prenatal care, parity and gravidity. CI – confidence interval, LGA – large for gestational age, GDM – gestational diabetes mellitus, HDP – hypertensive disorder of pregnancy, OR – odd ratio, SGA – small for gestational age.

After adjusting for confounding factors, maternal advanced age showed a statistically significant association with an higher likelihood of developing GDM (OR = 1.79; 95% CI = 1.75, 1.83) and HDP (OR = 1.93; 95% CI = 1.86, 2.01). Previous caesarean section was also linked with an increased risk of GDM (OR = 1.13; 95% CI = 1.11, 1.16) and HDP (OR = 1.24; 95% CI = 1.20, 1.28). There was also a positive correlation between GDM and the occurrence of LGA infants (OR = 1.32; 95% CI = 1.30, 1.35). Similarly, HDP was significantly associated with a higher risk of delivering SGA infants (OR = 3.93; 95% CI = 3.75, 4.12) (**Figure 4**).

### Mediating effect of GDM and HDP on the association between maternal advanced age and previous caesarean section, and risks of LGA and SGA

In terms of maternal advanced age, HDP emerged as a significant mediator in the risk of SGA, contributing to 68.96% of the mediation effect. Although there were substantial total and natural direct effects in the mediation of GDM on the risk of LGA, the natural indirect effect was not statistically significant. Further analyses showed that GDM played a significant mediating role in the link between previous caesarean section and LGA, accounting for 5.62% of the mediation effect. Similarly, HDP presented a mediating effect on the association with the risk of SGA, contributing to 4.49% of the mediation effect (**Table 2**, **Table 3**).

**Table 2:** Effect decomposition of the influence of advanced maternal age and potential mediators on fetal growth in live births in Southeast of China

Mediator	Total effect (95% CI)	Natural direct effect (95% CI)	Natural indirect effect (95% CI)	Mediated proportion, %
	**SGA**			
HDP	−0.00578 (−0.00757, −0.00398)*	−0.00976 (−0.01155, −0.00797)*	0.003984 (0.003778, 0.004189)*	68.96
	**LGA**			
GDM	0.004430 (0.04126, 0.04734)*	0.03880 (0.03574, 0.04186)*	0.005496 (−0.005113, 0.005879)†	12.41

CI – confidence interval, LGA – large for gestational age, GDM – gestational diabetes mellitus, HDP – hypertensive disorder of pregnancy, SGA – small for gestational age

*P < 0.001.

†*P* = 0.0711.

**Table 3:** Effect decomposition of the influence of previous caesarean section and potential mediators on fetal growth in live births in Southeast of China

Mediator	Total effect (95% CI)	Natural direct effect (95% CI)	Natural indirect effect (95% CI)	Mediated proportion, %
	**SGA**			
HDP	−0.01694 (−0.01860, −0.01529)*	−0.01770 (−0.01935, −0.01605)*	0.000760 (0.000605-0.000915)*	−4.49
	**LGA**			
GDM	0.04855 (0.04575, 0.05136)*	0.04582 (0.04302, 0.04863)*	0.002730 (0.002517, 0.002943)*	5.62

CI – confidence interval LGA – large for gestational age, GDM – gestational diabetes mellitus, HDP – hypertensive disorder of pregnancy, SGA – small for gestational age

*P < 0.001.

## DISCUSSION

Our primary objective was to investigate the mediating role of GDM and HDP in the relationship between maternal advanced age, previous caesarean section, and the likelihood of abnormal birth weight in women with singleton live births. The findings from our study showed significant links between maternal advanced age, previous caesarean section, GDM or HDP, and the risks of LGA and SGA. The mediation analysis, in turn, highlighted that the connections between maternal advanced age, previous caesarean section, and the risks of LGA and SGA were influenced by GDM or HDP. Specifically, HDP was found to mediate the association between maternal advanced age and the risk of SGA, while GDM mediated the relationship between previous caesarean section and the risk of LGA. Similarly, HDP played a mediating role in the link between previous caesarean section and the risk of SGA.

Fetal intrauterine growth is influenced by a complex interplay of factors including hormonal environment, genetic influences, and nutrient exchange between mother and fetus [43]. Maternal factors play a critical role in determining growth, including those such as maternal nutritional status, placental function, and metabolism, all of which can be impacted by pregnancy complications [44]. Various obstetric issues such as maternal age and previous caesarean sections have been linked with adverse outcomes [34,45,46]. Existing research has established a connection between maternal age and the risk of HDP [45,47], with HDP increasing the likelihood of giving birth to babies that are SGA [48,49]. We likewise found a significant association between maternal age and HDP, with HDP being a notable risk factor for SGA. Multiple studies have identified advanced maternal age as an independent risk factor for SGA [20,50–53]. A study of the Hokkaido birth cohort [48] explored the risk factors of HDP and the association between maternal characteristics and HDP on birth outcomes; the authors found that the odds between HDP and SGA was higher than that between maternal advanced age and HDP and between maternal advanced age and SGA. This suggests that HDP may have a more significant impact on fetal growth than maternal age [48]. Maternal advanced age was previously found to be associated with higher levels inflammatory markers, and oxidative stress, which may be used to explain the increased risk of HDP [54]. Furthermore, HDP may impede fetal growth through vascular mechanisms related to maternal fluid expansion [55]. A systematic review by Li and colleagues [56] showed that a SGA newborn is more likely to be delivered among women with HDP. However, the mediating role of HDP in the relationship between maternal age and SGA has not been extensively explored in prior studies. Here we found a significant mediating effect of HDP in the link between maternal age and SGA, highlighting on the complex relationship between maternal characteristics and pregnancy complications.

We observed a significant mediating role of HDP in the association between maternal advanced age and SGA, as well as the impact of changes in maternal characteristics on the heightened risk of maternal complications overall. Specifically, both maternal advanced age and previous caesarean section were found to be influenced by HDP in their relationship with SGA, with the latter further mediated by GDM in its association with LGA. This suggests that a history of caesarean section can have lingering effects on subsequent pregnancies, potentially leading to maternal and neonatal issues such as preeclampsia and SGA [57,58]. The negative impacts of previous caesarean section on birth weight have been documented in several studies [36,59,60]. Qin et al. [58] noted the path of suppression effects from previous caesarean section on birth weight, showing that previous caesarean section had negative effects on neonatal birth. It is widely recognised that GDM can lead to excessive fetal growth, as heightened glucose levels are transferred to the fetus, resulting in overgrowth [1,61]. A scarred uterus due to a history of caesarean section weakens the myometrium and vascularisation, potentially causing malnourishment in fetuses, leading to LGA and SGA babies. Furthermore, the compromised muscle layers and vascular degeneration also explain the heightened risks of GDM and HDP.

The results of our mediation analyses showed that HDP plays a mediating role in the impact of maternal advanced age and previous caesarean section on SGA risk, and the mediation effect of GDM on the association between previous caesarean section and LGA risk was also significantly. This approach allows us to gather insights about the potential contribution of GDM and HDP, although they may not be the only driver on fetal abnormal growth. These findings are meaningful to public health and in the clinical context. That is, fetal abnormal growth is mediated by GDM and HDP among pregnant women with advanced age or previous caesarean section. By studying the mechanisms through which these conditions affect fetal outcomes, we may better understand the underlying factors and offer opportunities to improve pregnancy outcomes. Combined with our results, this suggests that women who are of advanced age or have previously had caesarean section should actively and dynamically monitor the glucose and blood pressure level to reduce the risk of GDM and HDP and further fetal abnormal growth. Furthermore, by recognising that there are still excessive cases of fetal abnormal growth that are not attributed to GDM or HDP, we also recognise that there is still work to be done in understanding the underlying aetiology of these outcomes.

Our study has several strengths. First, the multicentric prospective cohort design with a substantial sample size provided strong statistical power to investigate the mediating effects of GDM and HDP on the linkage between maternal advanced age, previous caesarean section, and the risks of LGA and SGA. Additionally, our study explained the pathway of the connection between maternal advanced age, previous caesarean section, GDM, and HDP, as well as the risks of LGA and SGA, potentially aiding in the improvement of prevention strategies and offering guidance for managing pregnant women to lower the risk of abnormal birth weight. Nonetheless, our methodology also had some limitations. First, we cannot generalise our findings as our sample originated only from southeastern China. Other potential covariates of interest (geographic and cultural factors) were not well defined in our data source and could not be assessed, but should be quantified using other sources of data in future to expanding on findings and improve the broader applicability of the results. Second, we could not consider potential confounding factors like pre-pregnancy body-mass index (BMI), gestational weight gain, personal socioeconomic status, and occupation, potentially introducing bias in our results. The risk of GDM and HDP was previously found to be associated with high pre-pregnancy BMI and excessive gestational weight gain [62], both of which had also been linked to fetal overgrowth [63,64]. Personal socioeconomic status and occupation can also be associated with pre-pregnancy BMI and gestational weight gain; however, not adjusting for these factors possibly overstated our effect estimates, but was unlikely to have impacted the direction of relationship [65]. Third, as the mediation analysis tends to be under-powered, it can only prove a causal relationship to a certain extent, so this should ultimately be tested through experimental study designs. Also, with respect to LGA or SGA models, we did not have the information of the timing of GDM or HDP, which may occur after the diagnose of GDM or HDP, resulting in a misclassification exposure. Therefore, there is a need for further research integrating more potential factors in examining the mediating effect of GDM and HDP on the connection between maternal advanced age, previous caesarean section, and fetal birth weight.

## CONCLUSIONS

Based on our analyses, we propose that GDM and HDP could potentially act as mediators in the influence of maternal advanced age and previous caesarean section on the risks of LGA and SGA.
